# A translational in vivo model of trigeminal autonomic cephalalgias – therapeutic characterization with brainstem stimulation

**DOI:** 10.1186/1129-2377-14-S1-P36

**Published:** 2013-02-21

**Authors:** S Akerman, PR Holland, O Summ, MP Lasalandra, PJ Goadsby

**Affiliations:** 1 University of California, San Francsico, USA

## Introduction

Trigeminal autonomic cephalalgias (TACs) are highly disabling primary headaches that involve activation of trigeminovascular neurons and their reflex connection with the parasympathetic outflow to the cranial vasculature, via the superior salivatory nucleus (SuS) in the brainstem. Our understanding of TAC pathophysiology, and how and where treatments act, is hindered by a lack of animal models.

## Objectives

To validate an in vivo model of TACs that includes both trigeminal and autonomic responses using brainstem stimulation of the trigeminal-autonomic reflex.

## Methods

Rats were anesthetized with pentobarbitone (60 mgkg-1) and prepared for physiological measurement. Electrophysiological techniques were used to record neurons of the trigeminocervical complex, using SuS stimulation, to activate the trigeminal-autonomic reflex. We compared the affects of specific TAC treatments with those of similar class used for other primary headaches. We also looked at autonomic responses through blood flow observations around the lacrimal duct.

## Results

SuS stimulation resulted in two distinct populations of TCC neurons. Shorter latency neurons were unresponsive to 100% oxygen and the autonomic ganglion blocker, hexamethonium bromide. Longer latency responses were inhibited by oxygen (P < 0.05, n=20) and hexamethonium (P < 0.05, n=16). These longer latency responses were also preferentially inhibited by indomethacin and a triptan. Similarly 100% oxygen (P < 0.05, n=9) and hexamethonium (P < 0.05, n=7) inhibited evoked blood flow changes in the lacrimal duct. Likewise these responses were also inhibited by indomethacin and a triptan, but not naproxen or the CGRP receptor antagonist, olcegepant.

## Conclusion

This is the first in vivo characterization of both trigeminovascular and cranial autonomic manifestations present in TACs and demonstrates that brainstem activation may drive both sensory and autonomic symptoms. Both manifestations were specifically inhibited by highly effective TAC treatments, and some part of their locus of action is via the parasympathetic pathway.

## Competing interests

SA has consulted for Merck, PJG has consulted for Merck and Linde gases. OS has received grant funding from Merck.

